# The role of the TAPSE/sPAP ratio as a predictor of mortality in Pulmonary Arterial Hypertension: Its value for patient risk stratification

**DOI:** 10.1016/j.jhlto.2024.100168

**Published:** 2024-10-19

**Authors:** Paul Palacios-Moguel, Guillermo Cueto-Robledo, Héctor González-Pacheco, Jorge Ortega-Hernández, María Berenice Torres-Rojas, Dulce Iliana Navarro-Vergara, Marisol García-Cesar, Cinthia Alejandra González-Nájera, Carlos Alfredo Narváez-Oríani, Julio Sandoval

**Affiliations:** a Intensive Care Unit, American British Cowdray Medical Center, Mexico City; b Pulmonary Hypertension Clinic, General Hospital of Mexico Eduardo Liceaga, Mexico City; c Ignacio Chavez National Institute of Cardiology of Mexico, Mexico; d Cardiology department, Hospital General ISSSTE, Tacuba, Mexico City

**Keywords:** TAPSE/sPAP ratio, Pulmonary arterial hypertension, Right ventricular failure, Risk stratification, Ventriculo-arterial coupling

## Abstract

**Background:**

The tricuspid annular plane systolic excursion and systolic pulmonary artery pressure (TAPSE/sPAP) ratio has been proposed as an indicator of ventriculo-arterial coupling, predicting right ventricular failure (RVF) and mortality in patients with pulmonary arterial hypertension (PAH).

**Objective:**

To evaluate the usefulness of the TAPSE/sPAP ratio in predicting outcomes and improving risk stratification in patients with PAH.

**Methods:**

156 patients with PAH were included. Clinical, functional, echocardiographic, and haemodynamic variables, along with the TAPSE/sPAP ratio, were analysed based on etiological PAH subgroups and outcomes. Additional statistical measures, such as the area under the curve (AUC), net reclassification index (NRI), and integrated discrimination improvement, assessed the predictive ability of TAPSE/sPAP in combination with the ESC/ERS risk score, and other risk assessment strategies (COMPERA and Reveal Lite 2).

**Results:**

Most patients were female (86.5%), with a median age of 45.5 (IQR: 29–58) years. The TAPSE/sPAP ratio for the whole group was 0.26 (IQR: 0.190–0.347) mm/mmHg, which was similar among different aetiologies, but different between deceased and surviving patients (0.14 vs. 0.27 mm/mmHg, respectively, *P* < 0.001). A TAPSE/sPAP ratio <0.18 mm/mmHg independently predicted mortality (AUC: 0.859, 95% CI: 0.766– 0.952; *P* < 0.001). Integration with the ESC/ERS risk score improved predicted mortality (AUC: 0.87 vs. 0.75, *p* = 0.002) and risk stratification, reclassifying 14.28% of events and 36.92% of non-events, with an NRI of 39.4% (*P* < 0.001). Likewise, integration with other scores improved predicted ability of COMPERA and REVEA Lite2; COMPERA+TAPSE/sPAP (AUC: 0.837 vs 0.742; *p* = 0.005) and REVEAL Lite 2 +TAPSE/sPAP (AUC: 0.840 vs. 0.713; *p* < 0.001).

**Conclusions:**

A TAPSE/sPAP ratio <0.18 mm/mmHg predicts mortality in PAH. The combination of the TAPSE/sPAP ratio with the ESC/ERS risk score improved risk stratification, and reclassification emphasizing the potential of ESC/ERS+TAPSE/sPAP as a valuable tool for risk assessment and clinical decision-making in PAH patients. Integration of TAPSE/sPAP ratio with other scores (COMPERA and (REVEAL Lite 2) also improved the risk stratification and reclassification of these risk scores.

## Introduction

Pulmonary arterial hypertension (PAH) encompasses a group of diseases characterized by a broad spectrum of pulmonary vascular changes leading to elevated pulmonary artery pressures, right heart failure (RHF), and untimely death. Right ventricular function is the main determinant of the symptomatology and outcome of patients with PAH.^1^, ^2^, ^3^, ^4^, ^5^ Thus, assessing the function of the right ventricle (RV) in the setting of PAH is a matter of utmost importance.

While RV function can be assessed using indices such as cardiac index, RV ejection fraction (RVEF), tricuspid annular plane systolic excursion (TAPSE), and the right ventricular fractional area change, these parameters do not completely reflect how the RV adapts to the increase in afterload by increasing contractility to preserve ventricle-arterial (V-A) coupling and maintain blood flow.^6^, ^7^, ^8^, ^9^, ^10^

The TAPSE to systolic pulmonary artery pressure (TAPSE/sPAP) ratio, assessed through echocardiography, is suggested as a surrogate for the end-systolic elastance/arterial elastance (Ees/Ea) ratio determined by hemodynamics (RV-AP coupling).^6^, ^7^, ^8^, ^9^, ^10^, ^11^ This ratio is now incorporated into the risk stratification guidelines of the 2022 European Society of Cardiology (ESC)/European Respiratory Society (ERS) for pulmonary hypertension (PH).^12^ In the present study, we sought to determine the prognostic performance of the TAPSE/sPAP ratio along with the suggested 2015 ESC/ERS 3-strata model (categorizing risk as low, intermediate, or high) at the time of diagnosis^13^ in predicting outcomes and improving risk stratification and reclassification in patients with PAH (group 1). The prognostic performance of the TAPSE/sPAP ratio in combination with other risk stratification strategies such as the COMPERA (14) and the REVEAL Lite 2 (15), was also evaluated.

## Methods

A comparative, cross-sectional, and retrospective study was conducted in the Pulmonary Hypertension Clinic of a tertiary reference center for pulmonary hypertension in Mexico City. We included 156 patients with PAH prospectively enrolled to the institutional registry of PH between January 2013 and January 2023. PAH diagnosis was established in accordance with 2015 ESC/ERS guidelines for pulmonary hypertension.^13^ The diagnostic workup included right heart catheterization (RHC), and the diagnosis of PAH was confirmed by the exclusion of secondary causes of pulmonary hypertension and demonstration of a mean pulmonary artery pressure (mPAP) >25 mmHg at rest, pulmonary capillary wedge pressure (PCWP) <15 mmHg, and pulmonary vascular resistance >3 Wood units by RHC.^13^ All patients underwent right heart Doppler echocardiography within 3 days of RHC and received targeted therapy based on clinical evaluation and drug availability.

Doppler echocardiography was performed according to current Guidelines (16) by a single experienced cardiology echocardiographer, using Vivid E9 and Vivid S5 systems (GE Healthcare, Wauwatosa, WI). Tricuspid annular plane systolic excursion (TAPSE) was obtained by M-mode imaging in the apical four-chamber view centered on the right ventricle. The echo estimation of the sPAP was based on the sum of the peak velocity of tricuspid regurgitation (Bernoulli equation) and of the estimated central venous pressure obtained by inferior cava vein diameter and collapsibility. Long-term follow-up occurred through hospital visits or telephone contact. All efforts were made to establish the cause of any deaths. The ethics committee of the General Hospital of Mexico approved the study, and each participant gave informed consent upon admission.

### Data analysis

We analysed clinical, functional, echocardiography, and haemodynamic variables for the whole group and separately for subgroups of patients with PAH who died or survived long term. Categorical variables are presented as frequencies and percentages. Continuous variables were tested using the Kolmogorov–Smirnov test to determine their Distribution. Nonnormally distributed data were expressed as median interquartile range (IQR) and analyzed between groups with the nonparametric Mann Whitney U or Kruskal-Wallis test when 2 or >2 groups were compared, respectively. Normally distributed continuous variables were expressed as mean±SD, and comparisons were made with ANOVA tests. Significant differences between groups were identified using *X*_2_ or Fisher exact probability tests for categorical variables.

To determine optimal cut-off values for the TAPSE/sPAP ratio for predicting mortality, receiver operating characteristic (ROC) curve analysis was performed, and the exact cut-off value was determined using Youden’s index.^14^ Univariate analysis was used to examine the relationship between survival and selected demographic, medical history, laboratory, echocardiography, and haemodynamic variables. Odds ratios (ORs) with 95% confidence intervals (CIs) were calculated. A multivariable regression model with backward selection was used to adjust for potential confounders based on established associations between clinical, echocardiography, laboratory, and haemodynamic variables and mortality. Candidate covariates for multivariate analysis were selected from variables associated with mortality in univariate analysis. The Kaplan–Meier method was used to estimate overall survival. The date of RHC was used as the index for determining survival. Differences in survival curves for deceased and surviving patients according to TAPSE/sPAP cut-off values were tested by the log-rank procedure.

We compared the mortality estimates and risk discrimination of the ESC/ERS risk score; Comparative, Prospective Registry of Newly Initiated Therapies for Pulmonary Hypertension (COMPERA) (14) and also with those of the Registry to Evaluate Early and Long-Term PAH Disease Management (REVEAL), REVEAL Lite 2, (15) risk assessment strategies. Variables for risk stratification in the 2015 ESC/ERS guidelines were: WHO-FC, 6-min walk test distance (6MWD), and BNP, RAP, CI, and SvO2,^13^ COMPERA risk strategy included WHO functional class, 6-min walk test, BNP, RAP, CI, and SvO2. For each patient, the sum of all grades was divided by the number of available variables and rounded to the next integer to define the risk group. For the REVEAL Lite 2 risk assessment, variables included WHO functional class, systolic blood pressure, heart rate, 6-min walk distance, BNP, and eGFR. For comparison both in the COMPERA and in the REVEAL Lite 2, a three-category was computed in which patients in each strategy were classified as 1 =low risk, 2 =intermediate risk, and 3 =high-risk.

We used the cut-off value of the TAPSE/sPAP ratio (<0.180), obtained from the ROC analysis, for risk stratification according to TAPSE/sPAP ratio as follows: TAPSE/sPAP ratio <0.180 (value=1), TAPSE/sPAP ratio ≥0.180 (value=0). These values were integrated (added) to the scores of the ESC/ERS, COMPERA and in the REVEAL Lite 2 to obtain the ESC/ERS +TAPSE/sPAP, COMPERA + TAPSE/sPAP and REVEAL Lite 2 + TAPSE/sPAP stratification models. We used the DeLong test to compare AUCs between the stratification models.

For obtaining the net reclassification index (NRI), and integrated discrimination improvement (IDI) we estimated an ideal probability of death in our cohort (21.7% of predicted mortality) to divide the patients in low-intermediate risk (<21.7%) to those in high-risk categories (≥21.7%). All analyses were two-tailed, and values of *P* < 0.05 were considered significant. IBM SPSS Statistics (v. 20; IBM Corp., Armonk, NY) was used for the analyses.

## Results

From an initial evaluation of 168 patients, twelve were excluded due to incomplete data in seven patients, and due to a very low left ventricular ejection fraction (less than 40%) on echocardiography in five patients. One hundred and fifty-six patients were included. Demographic, functional, echocardiography, and haemodynamic characteristics of patients with PAH at the initial diagnostic RHC are summarized in Table 1. Most patients were female (86.5%), with a median age of 45.5 (IQR: 29–58) years. All of them had Hispanic ancestry. Etiological diagnoses included idiopathic PAH in 49 (31.4%) patients, PAH associated with congenital heart disease in 49 (31.4%), PAH associated with conective tissue disease in 47 (30.1%), and porto-PH in 11 (7%). All patients had PAH, with a median pulmonary artery pressure (median-PAP) of 58 (IQR: 38–65) mmHg, a normal PCWP of 5.0 (IQR: 5–5) mmHg, and an increased pulmonary vascular resistance index (PVRi) of 13.3 (IQR: 8–22.5) WU/m2. Most patients were in World Health Organization functional class I and II, and 47 (30.1%) were in class III and IV. 6MWD, was 342.8 ± 126.5 m. The median serum level of brain natriuretic peptide (BNP) was 144.5 pg/mL (IQR: 40.2–560), and the TAPSE/sPAP ratio for the whole group was 0.26 (IQR: 0.190–0.347) mm/mmHg. Table 1 also shows differences among etiological groups. Patients with PAH associated to congenital heart disease were younger and tended to be in a better functional class, whereas patients with portopulomary hypertension had lower median-PAP and PVRi. The TAPSE/sPAP ratio was similar among different aetiologies. Overall, the TAPSE/sPAP ratio in the group correlated significantly (p = 0.001) with the haemodynamic parameters obtained from RHC. TAPSE/sPAP correlated negatively with right atrial pressure (r = −0.430), mPAP (r = −0.430), and PVRi (r = −0.464), and positively with cardiac index (r = 0.399).

**Table 1:** Demographic, clinical, functional, laboratory, echocardiography, and haemodynamic characteristics of patients with PAH.

Variable	All PAH patients (n = 156)	IPAH (n = 49)	PAH-CHD (n = 49)	PAH-CTD (n = 47)	Portal Hypertension (n = 11)	p value
BMI, median (IQR), kg/m^2^	26.4 (22.5–30.5)	28.2 (23.6–34.7)	26.4 (21.9–30.2)	25.7 (21.5–28.3)	28.1 (24.6–31.7)	0.026
WHO FC ≥ III, n (%)	47 (30.1)	17 (34.7)	10 (20.4)	17 (36.1)	3 (27.2)	0.315
6MWT mean± SD (m)	342.8 ± 126.5	321.7 ± 144.6	365 ± 119.5	349 ± 114	309 ± 114	0.351
BNP, median (IQR), pg/mL	144.5 (40.2–560)	156.4 (48.5–581.3)	160.2 (48.3–650)	70 (25–653)	181.6 (94.4–266)	0.199
Creatinine, median (IQR), mg/dL	0.80 (0.69–1.0)	0.80 (0.70–1.1)	0.82 (0.70–1.0)	0.80 (0.60–0.90)	0.90 (0.83–1.2)	0.109
LVEF, median (IQR) (%)	65 (60–70)	65 (60–70)	65 (55–72)	66 (62–71)	67 (65–72)	0.282
RAA, median (IQR), cm^2^	20 (16–28)	22.5 (18–30)	22 (18.2–29.2)	17.5 (13.5–22)	19 (15.2–21.75)	0.001
SPAP-e, median (IQR) (%), mmHg	74 (60–90)	75 (61.5–97.5)	75 (62.5–88.5)	68 (51–82)	78 (54–83)	0.093
TAPSE, median (IQR), mm	18 (16–22)	19 (15–21.5)	18 (16–21.5)	19 (16.8–22)	22 (19–25)	0.046
RVFAC, median (IQR) (%)	36.5 (31–42)	36 (30.5–42.5)	35 (28–40)	37 (33–43)	38 (35–51)	0.132
TAPSE/sPAP ratio, median (IQR), mm/mmHg	0.26 (0.19–0.34)	0.24 (0.16–0.31)	0.25 (0.18–0.32)	0.27 (0.20–0.40)	0.30 (0.26–0.40)	0.077
HR, median (IQR), beats/min	84 (74–93.5)	80 (73.5–92.5)	86.5 (75–90.2)	86 (76–95)	78 (69–88)	0.371
RAP, median (IQR) (mmHg)	6.0 (3.0–10.0)	7.0 (5.0–12.0)	8.0 (5.0–11.0)	6.0 (2.0–9.0)	3.0 (2.0–5.0)	0.003
SPAP, median (IQR) (mmHg)	80 (60–104)	89 (75–105)	83 (64.5–113.5)	64 (47–95)	50 (42–79)	< 0.001
mPAP, median (IQR) (mmHg)	52 (38–65)	56 (45–68)	56 (41–79.5)	42 (30–58)	38 (23–46)	< 0.001
PCWP, median (IQR) (mmHg)	5.0 (5.0–5.0)	5.0 (5.0–7.0)	5.0 (5.0–5.0)	5.0 (5.0–5.0)	5.0 (5.0–5.0)	0.051
CI, median (IQR) (L/min/m^2^)	3.2 (2.7–4.0)	3.0 (2.45–3.55)	3.4 (2.75–4.30)	3.4 (2.6–4.0)	3.5 (3.1–4.1)	0.079
SvO_2_%, median (IQR), %	71 (63.5–76)	69 (62–74)	75 (65.5–80)	72 (61–75)	69 (67–75)	0.077
PVR, median (IQR), WU	7.8 (4.5 −13.9)	10.1 (6,3 −15.6)	8.5 (4.9 −15.2)	6.7 (3.8 −11.3)	4.5 (3.8 −5.9)	0.003
PVRi, median (IQR), WU m^2^	13.3 (8.0–22.5)	17.4 (11.9–26.2)	15.8 (8.6–23.1)	11 (7.0–17.4)	7.7 (5.8–10.8)	0.001
SVRi, median (IQR), m^2^	26.5 (21–33)	28.8 (25.1–34.4)	22.8 (19.6–33.1)	26.5 (20.7–33.6)	26.2 (22.4–31)	0.037

Abbreviations: PAH: pulmonary arterial hypertension; IPAH: idiopathic PAH; PAH-CHD: PAH associated with congenital heart disease; PAH-CTD: PAH associated with collagen tissue disease; Po-Pul PAH: portopulmonary PAH; BMI: body mass index; WHO FC: World Health Organization Functional Class; 6MWT: six-minute walk test; BNP: brain natriuretic peptide; LVEF: left ventricular ejection fraction; RAA: right atrial area; SPAP-e: systolic pulmonary artery pressure by echo; TAPSE: tricuspid annulus plane systolic excursion; RVFAC: right ventricular fractional area change; RAP: right atrial pressure; SPAP: systolic pulmonary artery pressure; mPAP: mean pulmonary artery pressure; PCWP: pulmonary capillary wedge pressure; CI: cardiac index; SvO_2_: mixed-venous oxygen saturation; PVR: pulmonary vascular resistance; WU: Wood units; PVRi: pulmonary vascular resistance index; SVRi: systemic vascular resistance index. SD: standard deviation.

**Figure 1:**
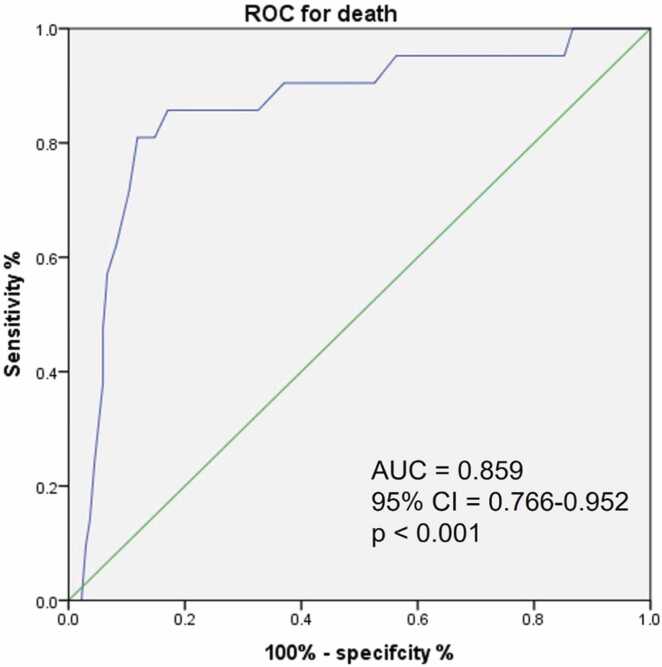
Receiver-operating characteristic (ROC) curve showed significant performance (AUC: 0.859) for the accuracy of TAPSE/sPAP ratio < 0.180 in predicting death in patients with PAH.

Differences between deceased and surviving patients are shown in Table 2. There were significant differences in baseline functional class, risk stratification, BNP, creatinine levels, TAPSE, and pulmonary haemodynamics. The TAPSE/sPAP ratio also differed between deceased and surviving patients (0.14 (IQR: 0.11–0.17) vs. 0.27 (IQR: 0.22–0.36) mm/mmHg, respectively, P < 0.001). To further explore the role of the TAPSE/sPAP ratio, we determined the optimal cut-off for predicting mortality by performing ROC curve analysis (AUC: 0.859; 95% CI: 0.766–0.952; P < 0.001), and an exact cut-off value of 0.18 mm/mmHg was determined using Youden’s index (Figure 1).

**Table 2:** Clinical, functional, echocardiography, and haemodynamic characteristics of dead vs. surviving patients with PAH.

		Dead (n = 21)	Alive (n = 135)	P value
Age, median (IQR) (years)		45 (36.0–60.0)	46 (29.0–58.0)	0.682
Female, n (%)		16 (76.2)	119 (88.1)	**0.135**
BMI, median (IQR), kg/m^2^		26.0 (21.5–31.3)	26.6 (22.7–30.4)	0.580
WHO Functional Class, n (%)	I	1 (4.8)	36 (26.7)	0.025
	II	9 (42.9)	63 (46.7)	
	III	8 (38.1)	31 (23.0)	
	IV	3 (14.3)	5 (3.7)	
Risk stratification (%) **ESC/ERS**	Low	3 (14.3)	85 (63.0)	< 0.001
	Intermediate	17 (81.0)	50 (37.0)	
	High	1 (4.8)	0 (0.0)	
Risk stratification (%) **COMPERA 1.0**	Low	7 (33.3)	107 (79.3)	< 0.001
	Intermediate	11 (52.4)	27 (20.0)	
	High	3 (14.3)	1 (0.7)	
Risk stratification (%) **REVEAL Lite 2**	Low	3 (14.3)	71 (52.6)	0.005
	Intermediate	8 (38.1)	28 (20.7)	
	High	10 (47.6)	36 (26.7)	
6MWT, median (IQR), m		309.0 (190.5–371.5)	370.0 (250–445)	0.084
BNP, median (IQR), pg/mL		705.8 (161.3–1214)	99 (34–387)	< 0.001
Creatinine, median (IQR), mg/dL		0.92 (0.80–1.28)	0.80 (0.67–0.95)	0.007
LVEF, median (IQR) (%)		65 (61–74.5)	65 (59–70)	0.458
RAA, median (IQR), cm^2^		22 (19–29)	20 (16–27)	0.216
SPAP-e, median (IQR), mmHg		96 (85–113)	70 (55–83)	**< 0.001**
TAPSE, median (IQR), mm		15 (12–17)	19 (17–22)	**< 0.001**
RVFAC, median (IQR) (%)		34 (28–41)	37 (32–43)	0.227
TAPSE/sPAP ratio, median (IQR), mm/mmHg		0.14 (0.11–0.17)	0.27 (0.22–0.36)	**< 0.001**
TAPSE/sPAP ratio, < 0.180, n (%)		17 (80.9)	16 (11.8)	**< 0.001**
HR, median (IQR), beats/min		85 (73–97)	84 (74–93)	0.963
RAP, median (IQR) (mmHg)		12 (9–14.5)	6 (3–9)	**< 0.001**
SPAP, median (IQR) (mmHg)		90 (81.5–125)	76.3 (57.0 −101)	**0.002**
mPAP, median (IQR) (mmHg)		62 (49.5–77)	49 (36–63)	**0.004**
PCWP, median (IQR) (mmHg)		5 (5–5)	5 (5–5)	0.679
CI, median (IQR) (L/min/m^2^)		2.7 (1.9–3.3)	3.3 (2.9–4.0)	**0.001**
PVR, median (IQR), WU		15 (10.1 −24.05)	6.8 (4.4 −11.4)	**< 0.001**
SvO_2_%, median (IQR), %		62 (57.5–72)	72 (65–76)	**0.013**

Abbreviations. As in Table 1.

The unadjusted mortality rate for the 156 patients was 13.5% (21 patients). Causes of death included heart failure due to disease progression in 13 (62%), bowel occlusion in 1 (5%), COVID-19 in 1 (5%), and sudden or unknown in 6 (28%) patients. Table 3 shows the results of the univariate and multivariate analyses of variables associated with mortality.

**Table 3:** Independent predictors of mortality by binary regression.

	**Unadjusted**			**Adjusted**		
	**OR**	**95% CI**	**P**	**OR**	**95% CI**	***P***
Age	1.005	0.978–1.032	**0.713**			
Gender, female	0.430	0.139–1.334	**0.144**			
Functional Class	2.347	1.315–4.188	**0.004**			
WHO Functional Class ≥ III	3.025	1.185–7.724	**0.021**			
Risk stratification intermediate/high	10.20	2.861–36.364	**< 0.001**	3.622	(0.84 −15.55)	0.084
BMI	0.981	0.908–1.060	**0.622**			
BNP	1.001	1.000–1.001	**0.072**			
Creatinine	3.300	1.001–10.878	**0.050**			
LVEF, median (IQR) (%)	1.022	0.971–1.076	**0.405**			
RAA	1.005	0.963–1.049	**0.819**			
TAPSE	0.789	0.692–0.899	**< 0.001**			
RVFAC	0.974	0.926–1.023	**0.294**			
sPAP	1.039	1.020–1.059	**< 0.001**			
TAPSE/sPAP ratio	0.000	0.000–0.000	**< 0.001**			
TAPSE/sPAP ratio, ≤ 0.18 mm/mmHg	31.609	9.447–105.76	**< 0.001**	**13.10**	(3.54 −48.41)	**< 0.001**
6MWT	0.997	0.994–1.001	**0.139**			
RAP	1.302	1.162–1.460	**< 0.001**	1.148	(0.99 −1.32)	0.061
mPAP	1.032	1.009–1.055	**0.005**			
CI	0.417	0.238–0.730	**0.002**			
PVRi	1.069	1.032–1.107	**< 0.001**			
SVRi	1.064	1.024–1.107	**0.002**			
SvO2%	0.956	0.924–0.989	**0.009**			

Abbreviations. As in Table 1.

**Figure 2:**
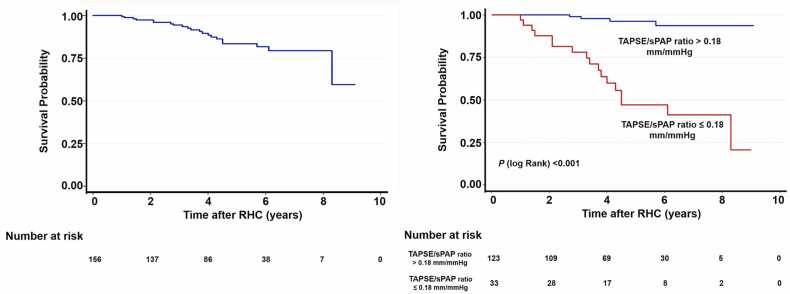
Kaplan–Meier survival estimates for the whole group of patients with PAH (left panel). Right panel: survival estimates according to a TAPSE/sPAP ratio above or below 0.180 at baseline. Patients with a TAPSE/sPAP ratio above 0.180 had significantly better survival than those with a TAPSE/sPAP ratio below 0.180 (median survival 8.8 (95% CI: 8.52–9.08 months) vs. 5.53 (95% CI: 4.46–6.60 months), respectively, Chi-squared log-rank 43.7; *P* < 0.001).

Only a TAPSE/sPAP ratio below 0.180 remained an independent predictor of mortality (OR: 13.10, 95% CI: 3.548–48.411, P < 0.001). Patients were followed for 4.31 years from RHC. Figure 2 shows the Kaplan–Meier survival estimates for the whole group and for patients with a TAPSE/sPAP ratio below or above 0.18 mm/mmHg at baseline.

**Figure 3:**
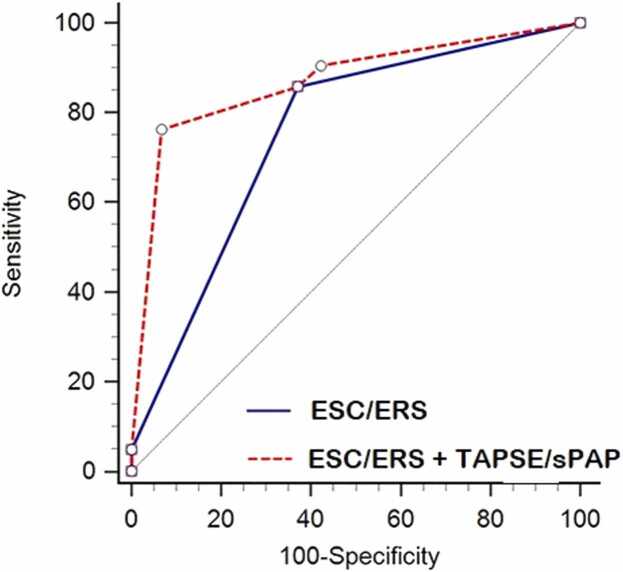
The area under the receiver operating characteristic curve (AUC) for ESC/ERS+TAPSE/sPAP and ESC/ERS risk score alone and their performance in predicting death in patients with PAH.

The evaluation of ESC/ERS+TAPSE/sPAP added predictive ability compared with the ESC/ERS risk score alone. In AUC analysis, ESC/ERS+TAPSE/sPAP demonstrated a significantly higher AUC compared with the ESC/ERS risk score alone (0.75 vs. 0.87), with a difference of 0.126 (95% CI: 0.0592–0.193; P < 0.001) (Figure 3**).** Compared also by DeLong test the addition of TAPSE/sPAP improved the pre-existing risk stratification in COMPERA and REVEAL Lite2 risk scores; COMPERA+TAPSE/sPAP (AUC: 0.837 vs 0.742; p = 0.005) and REVEAL Lite 2 +TAPSE/sPAP (AUC: 0.840 vs. 0.713; p < 0.001).

(Figure 4).

**Figure 4:**
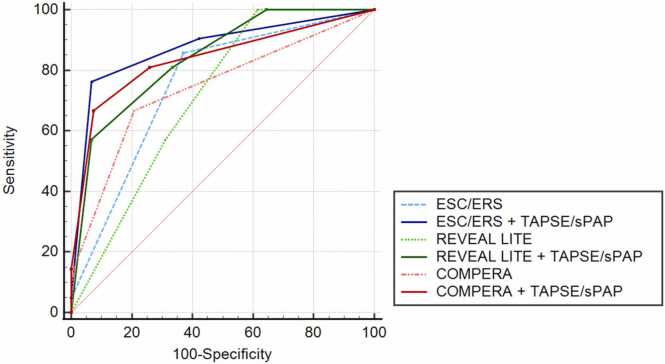
Impact of the integration of the TAPSE/SPAP risk score to other risk scores categories. In AUC analysis, ESC/ERS+TAPSE/sPAP demonstrated a significantly higher AUC compared with the ESC/ERS risk score alone (0.75 vs. 0.87; p < 0.001). COMPERA+TAPSE/sPAP (AUC: 0.837 vs 0.742 in the COMPERA alone; p = 0.005) and REVEAL Lite 2 +TAPSE/sPAP (AUC: 0.840 vs. 0.713 in the Reveal Lite 2,0 alone; p < 0.001), indicating that adding the TAPSE/sPAP risk score to the other risk scores provides superior discriminatory power in distinguishing the outcome.

**Figure 5:**
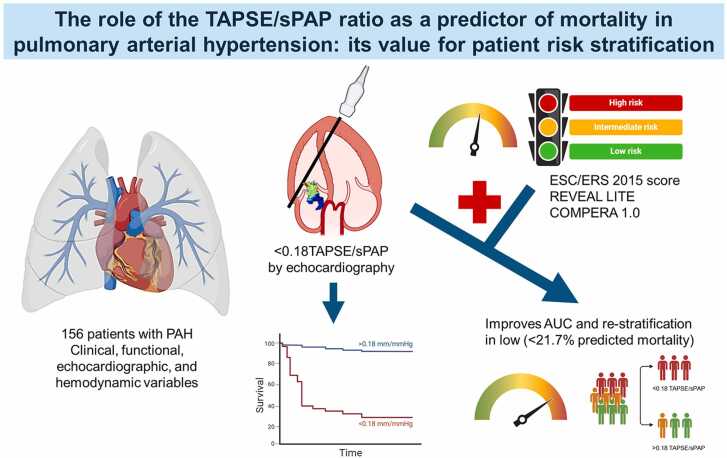
Central illustration. Proposed integration of the TAPSE/sPAP ratio risk score to other risk score strategies. Once the cut-off values of the TAPSE/sPAP radio are established in the PAH population under study (Low and high risk), this value could be integrated (added) to the risk score values of the ESC/ERS, COMPERA, and REVEAL Lite 2, risk stratification values. The TAPSE/sPAP ratio below < 0.18 mmHg in our population correctly differentiates mortality and its addition upgrades the pre-existing scores.

Net reclassification analysis was performed using an ideal cut-off mortality 21.7% for our cohort to divide in high- and low/intermediate -risk setting for the scores (Table 4). In the ESC/ERS risk score and the ESC/ERS+TAPSE/sPAP of the 21 patients who died, classification improved in 3 patients using the ESC/ERS+TAPSE/sPAP model. The NRI in all subjects was 39.4% (P < 0.001), correctly reclassifying 30.3% of non-events (P < 0.001). When applied to COMPERA the NRI proportion was 20.84% (P = 0.011), correctly reclassifying 30% (P < 0.001) of non-events. Lastly, in REVEAL Lite 2 the addition of TAPSE/sPAP NRI in all subjects was 24.44% (P < 0.001), but this correctly reclassified 24% of non-events (P < 0.001, Table 4). Thus, addition +TAPSE/sPAP significantly improved the identification of patients at low-intermediate risk of death (non-events) when compared with the ESC/ERS, COMPERA 1.0 and REVEAL Lite 2 risk score alone.

**Table 4:** Risk reclassification tables for different risk scores.

	**ESC/ERS+TAPSE/sPAP ratio**		
**ESC/ERS risk score**	Low-intermediate (<21.7%)	High risk (≥21.7%)	Proportion reclassified %
***Event***			
Low-intermediate (<21.7%) n = 3	0	3	100
High risk (≥21.7%) n = 18	0	18	0
***No events***			
Low-intermediate (<21.7%) n = 85	78	7	8
High risk (≥21.7%) n = 50	41	9	82
***Overall***			
Low-intermediate (<21.7%) n = 88	78	10	11
High risk (≥21.7%) n = 68	41	27	60
NRI (Categorical): 0.3947; 95% CI 0.2195–0.57; P < 0.001. NRI (Continuous): 1.763; 95% CI 1.6539–1.872; P < 0.001. IDI: 0.4307; 95% CI 0.3795–0.4818; P < 0.001.			

	**COMPERA+TAPSE/sPAP ratio**		

**COMPERA 1.0 risk score**	Low-intermediate (<21.7%)	High risk (≥21.7%)	Proportion reclassified %

***Event***			
Low-intermediate (<21.7%) n = 3	3	0	0
High risk (≥21.7%) n = 18	2	16	11
***No events***			
Low-intermediate (<21.7%) n = 85	85	0	0
High risk (≥21.7%) n = 50	41	9	18
***Overall***			
Low-intermediate (<21.7%) n = 88	88	0	0
High risk (≥21.7%) n = 68	43	25	63
NRI (Categorical): 0.20847; 95% CI 0.0609–0.356; P = 0.011. NRI (Continuous): 1.33439; 95% CI 0.9789–1.6899; P < 0.001. IDI: 0.28535; 95% CI 0.1934–0.3773; P < 0.001.			

	**REVEAL+TAPSE/sPAP ratio**		

**REVEAL 2.0 risk score**	Low-intermediate (<21.7%)	High risk (≥21.7%)	Proportion reclassified %

***Event***			
Low-intermediate (<21.7%) n = 9	9	0	0
High risk (≥21.7%) n = 12	0	12	0
***No events***			
Low-intermediate (<21.7%) n = 93	93	0	0
High risk (≥21.7%) n = 42	33	9	24
***Overall***			
Low-intermediate (<21.7%) n = 74	102	0	0
High risk (≥21.7%) n = 82	33	21	21
NRI (Categorical): 0.24444; 95% CI 0.1719–0.3169; P < 0.001. NRI (Continuous): 0.95026; 95% CI 0.5154–1.3851; P < 0.001. IDI: 0.19414; 95% CI 0.1003–0.2879; P < 0.001.			

## Discussion

Results from the current study confirm and extend previous observations that the simplified, echocardiographic approach of estimating RV-arterial coupling in patients with PAH by the TAPSE/sPAP ratio is very useful in the initial patient evaluation for prognostic purposes.^11^, ^15^, ^16^, ^17^, ^18^, ^19^, ^20^, ^21^, ^22^, ^23^ It correlates with the haemodynamic parameters of the RV from RHC and is clearly linked with long-term prognosis. Our results show that the TAPSE/sPAP ratio was similar among the different aetiologies of PAH, reflecting that RV function/dysfunction is an independent determinant of survival in patients with any form of PAH. Finally, through statistical analysis, our study demonstrates that the addition of the TAPSE/sPAP ratio category to the ESC risk score significantly improves the discrimination of mortality over the ESC/ERS risk score alone. The NRI demonstrated a reclassification of 39.4% in all subjects. Thus, ESC/ERS+TAPSE/sPAP significantly improved the identification of patients, reclassifying them to a lower risk of death when compared with the ESC/ERS risk score. Results in the present study also show that the addition of the TAPSE/sPAP ratio category to other risk stratification strategies such the COMPERA or the Reveal Lite 2 scores also improves the identification of patients. Improving stratification of risk at baseline in patients with PAH is important as it may influence our clinical decision-making regarding intensity of the initial treatment. It should, noted, however, that the population in the present study resembles more that of other Latin-American countries concerning PAH etiology (27, 28) and differs from that in North American and European registries. Accordingly, results from our study might apply to populations in some Latin American countries but not necessarily to other Western registries/populations.

RV function can be assessed using indices such as cardiac index and RVEF; however, these parameters are affected by RV preload and afterload and are not ideal indicators of RV intrinsic function.^6^, ^7^, ^8^, ^9^, ^10^ Ventricular elastance, calculated from pressure– volume loop analysis, is the reference index for RV function.^9^ RV Ees and end-diastolic elastance representing RV intrinsic systolic and diastolic function, respectively. In addition, Ees divided by Ea is a parameter of RV systolic function in relation to afterload, or pulmonary ventricle-artery (RV-PA) coupling.^7^, ^9^, ^10^, ^11^ Ideally, these indices can assess RV function. However, they are not widely used because specific pressure and conductance catheters and invasive procedures are required.^9^, ^23^ The Ees/Ea ratio (RV-PA coupling), considered the gold standard metric, can be estimated by calculating the individual components, Ees and Ea, from non-invasive images such as magnetic resonance imaging or echocardiography.

Recently, the TAPSE/sPAP ratio, measured by echocardiography, has been proposed as a surrogate for the invasive and demanding measurement of the Ees/Ea ratio by haemodynamics (RV-PA coupling), and several studies have supported its use to assess RV function in PH associated with heart failure^11^, ^15^, ^16^ as well as in PAH.^17^, ^18^, ^19^, ^20^, ^21^, ^22^, ^23^ In a retrospective study of 334 patients with heart failure, Guazzi et al.^11^ showed that a TAPSE/sPAP ratio < 0.36 mm/mmHg was associated with increased cardiac-related mortality (HR: 10.4, P < 0.001) and proposed that this TAPSE vs. sPAP relationship could be a step forward for a more efficient RV function evaluation, unaffected by the quality of LV dysfunction. This finding was confirmed by Gorter et al. in a prospective study of 102 patients in the same heart failure population.^15^

In a prospective, specific study for PAH, Tello et al.^19^ described 52 patients with PAH and chronic thromboembolic hypertension and showed a significant correlation of the TAPSE/sPAP ratio and final RV elastance assessed by RHC, confirming the TAPSE/sPAP ratio as an independent predictor of V-a coupling. Of all the surrogates evaluated, only the TAPSE/sPAP ratio emerged as an independent predictor of Ees/Ea (multivariate OR: 18.6; 95% CI: 0.8–96.1; P = 0.08). In ROC analysis, a TAPSE/sPAP cut-off of 0.31 mm/mm Hg discriminated RV arterial uncoupling (Ees/Ea < 0.805). Patients with TAPSE/sPAP < 0.31 mm/mm Hg had an increase in mortality.^19^

The usefulness of the TAPSE/sPAP ratio for assessing RV arterial uncoupling and outcomes in the setting of PAH has been confirmed.^17^, ^18^, ^19^, ^20^, ^21^, ^22^, ^23^ In a German population of 290 patients with PAH,^18^ stratified by tertile of TAPSE/sPAP (low: <0.19 mm/mmHg; middle: 0.19–0.32 mm/mmHg; high: >0.32 mm/mmHg), patients in the low tertile showed significantly compromised haemodynamic, functional, and echocardiographic status. In all multivariate models, the TAPSE/sPAP ratio remained independently associated with overall mortality. Kaplan–Meier analyses showed better overall survival in the middle and high tertiles vs. the low tertile.^18^ The prognostic usefulness of the TAPSE/sPAP has been also demonstrated in patients with PAH associated with collagen tissue disease.^24^

All These findings provide some validation of TAPSE/sPAP as a surrogate to invasively measure Ees/Ea for RV-PA coupling and suggest it is a reliable, non-invasive method to determine RV-PA coupling in patients with PH. However, whether this can be extrapolated to patients with different PH aetiologies, as well as to different stages of severity of PAH, is unknown.^25^ In the study of Gerges et al.,^26^ performed in patients with combined pre- and postcapillary PH and idiopathic PAH, the TAPSE/sPAP ratio for patients with idiopathic PAH was 0.14 ± 0.11, and there was no correlation with Ees/Ea. One possible explanation for this finding, offered by Bashline and Simon,^25^ is that TAPSE tends to reach a certain minimum despite the progression of RV dysfunction, while sPAP may actually, decrease as RV contractility decreases with the potential to overestimate the TAPSE/sPAP in certain situations. Another factor that may contribute is the degree of tricuspid regurgitation. It may falsely increase TAPSE when severe tricuspid reflux is found.

Our study included patients with different aetiologies of PAH, yet our results showed that the TAPSE/sPAP ratio was similar in all PAH aetiologies and that TAPSE/sPAP ratio < 0.18 mm/mmHg remained an independent predictor of mortality. This cut-off value is very similar to that reported in the new 2022 ESC/ERS Guidelines for PH (TAPSE/sPAP <0.19 mm/mmHg) in the evaluation of high-risk patients with PAH.^12^ Thus, it appears that, in our study and most others,^17^, ^18^, ^19^, ^20^, ^21^, ^22^, ^23^, ^24^ the TAPSE/sPAP ratio is a good predictor of mortality in PAH.

A combination of the TAPSE/sPAP ratio, along with other non-invasive parameters, may improve the assessment of the risk of short-term mortality in patients with PAH at baseline or at follow-up (32−33). Using a different methodology and risk score categories, Vicenzi et al.^27^ described the added value of the TAPSE/sPAP ratio, along with other parameters of RV function to improve the risk stratification of patients with PAH. In our study, statistical analyses demonstrated that adding the TAPSE/sPAP ratio category to the ESC risk score significantly improved the discrimination of mortality over the ESC risk score alone, as evidenced by improvement in the C-statistic and NRI. Assessing the TAPSE/sPAP ratio in the context of well-established clinical risk scores, such as the ESC risk score, allows the identification of subgroups, such as patients with a low TAPSE/sPAP ratio at high risk of death, and highlights the high TAPSE/sPAP ratio as a low mortality risk, regardless of ESC risk score values.

The proposed echocardiographic estimation of RV ventricular–arterial coupling offers an easy, reliable, and non-invasive prognostic parameter for a comprehensive assessment of haemodynamic adaptation in patients with PAH at baseline.^28^, ^29^ Accordingly, the inclusion of the TAPSE/sPAP ratio in the risk stratification of PAH in the 2022 ESC/ERS Guidelines for PH^12^ appears justified.

Also, the addition +TAPSE/sPAP significantly improved the identification (reclassification) of patients at low risk of death (non-events) when compared with the ESC/ERS, COMPERA 1.0 and REVEAL Lite 2 risk score alone (Table 4). Net reclassification analysis was performed using an ideal cut-off mortality 21.7% for our cohort to divide in high- and low-intermediate risk setting for the scores. It is interesting that the ideal cut-off value of 21.7% in our study resembles the new ideal cut-off value of mortality in the new 2022 PAH ESC guidelines of high risk= 20%. (12).

## Limitations

Limitations of the current study include the retrospective evaluation of patients, the limited number of events, the sample size being insufficient for robust analyses, such as multiple comparisons among etiological subgroups and, as we do not have an accurate and complete follow-up, the impossibility to assess the impact of specific PAH therapy on RV function and long-term survival.

## Conclusions

The echocardiographic estimation of the TAPSE/sPAP ratio offers an easy, reliable, and noninvasive prognostic parameter for the comprehensive assessment of haemodynamic adaptation in PAH, especially in a high-risk population, to predict a non-event in patients with adequate coupling. This ratio is significantly associated with outcomes and haemodynamics, irrespective of PAH etiology. In addition, the TAPSE/sPAP ratio improves risk stratification, enhancing the identification of patients at low-intermediate risk of death compared with the ESC/ERS, COMPERA and REVEAL Lite2 risk scores. If confirmed in a larger, non-Latino data registry, our results with the use of the TAPSE/sPAP ratio could open an avenue for a new risk stratification strategy.

## Funding statement

No funding.

## Conflict of Interest

Nothing to disclose.
